# Mapping the Availability of Rehabilitation Providers Using Public Licensure and Population Data for a Geographic Information System–Based Approach to Workforce Planning: Cross-Sectional Feasibility Study

**DOI:** 10.2196/85025

**Published:** 2025-12-23

**Authors:** Madeline Ratoza, Rupal M Patel, Julia Chevan, Wayne Brewer, Katy Mitchell

**Affiliations:** 1 University of St. Augustine for Health Sciences Austin, TX United States; 2 Texas Woman's University Houston, TX United States; 3 Springfield College Springfield, MA United States; 4 Texas Woman's University Denton, TX United States

**Keywords:** geographic information systems, rehabilitation workforce, spatial analysis, workforce planning, health service access

## Abstract

**Background:**

Access to rehabilitation services is a critical yet under-studied dimension of health equity. Among the 6 domains of access, health care provider availability, defined as the presence of sufficient health care providers to meet population needs, is particularly underexplored in rehabilitation professions such as physical and occupational therapy. Current data reporting often lacks the geographic granularity required for effective workforce planning.

**Objective:**

The purpose of this study was to demonstrate the feasibility of mapping rehabilitation provider availability at the census tract level using geographic information systems and integrating public licensure and population data to inform equitable workforce planning.

**Methods:**

A descriptive, cross-sectional study was conducted using publicly available state licensure data for physical and occupational therapists and demographic data from the American Community Survey. Residential addresses of rehabilitation providers were geocoded and matched to 2020 census tracts. Population-to-provider ratios were calculated and mapped using choropleth and bivariate mapping techniques. Population-to-provider ratios were calculated per tract and summarized overall and by rurality using 2020 Rural-Urban Commuting Area (RUCA) codes (urban: RUCA of 1-3; rural: RUCA of ≥4). The spatial dependence of ratios was tested using a spatial autocorrelation statistic, the global Moran *I*, in ArcGIS Pro using edge contiguity neighbors and row standardization.

**Results:**

Across 6896 tracts, ratios ranged from 4.5 to 11,147 persons per provider (median 1131, IQR 537-2501). By rurality, urban tracts (n=5734, 83.1%) had a median ratio of 1141 (IQR 2054), and rural tracts (n=1162, 16.9%) had a median ratio of 1093 (IQR 1690), indicating a broadly similar central tendency with somewhat greater variability in urban areas. The population-to-provider ratio exhibited significant positive spatial autocorrelation (global Moran *I*=0.305; *Z*=40.28; *P*<.001), consistent with clustered pockets of high and low availability rather than random dispersion.

**Conclusions:**

A replicable geographic information system protocol can integrate licensure and demographic data to produce interpretable population-to-provider metrics and spatial diagnostics at the census-tract level. In Texas, rehabilitation workforce availability is spatially clustered and not explained solely by an urban-rural divide, underscoring the value of small-area mapping for equitable workforce planning and policy decisions.

## Introduction

Access to health care is a key social determinant of health that directly influences outcomes across populations [1-6]. Within rehabilitation professions such as physical therapy (PT) and occupational therapy (OT), access is shaped by a combination of system-level and individual-level factors [7,8]. The concept of access has been widely studied and is frequently organized around the theory of access by Penchansky and Thomas [7], which outlines 6 interrelated dimensions: availability, accessibility, accommodation, affordability, acceptability, and awareness [8]. Among these, availability, or the extent to which a sufficient rehabilitation workforce is geographically and operationally present to meet population needs, is an underexamined dimension. Because rehabilitation care typically involves multiple visits across an episode of care, rehabilitation provider availability is essential to ensuring consistent, effective treatment [9].

The distribution of rehabilitation providers has direct implications for health outcomes. Limited availability can contribute to preventable decline in function, increased pain, and prolonged dependence on higher levels of care. The World Health Organization’s Rehabilitation 2030 initiative highlights this concern, identifying a profound unmet global need for rehabilitation [8]. The 2019 Global Burden of Diseases, Injuries, and Risk Factors study estimated that 2.41 billion individuals have conditions that would benefit from rehabilitation, which contributed to a 69% increase in years lived with disability since 1990 [10,11]. In North and South America, 1 in 3 people could benefit from rehabilitation, and in the United States, approximately 5 in 11 individuals have conditions requiring these services [10,11]. This growing need highlights the importance of equitable workforce distribution as a prerequisite for access to care.

Despite the centrality of provider availability to access, rehabilitation disciplines such as PT and OT are not included in federal shortage designation frameworks such as health professional shortage areas or medically underserved areas and populations [12-15]. These designations, which are used for primary care, dentistry, mental health, and maternity care, rely on population-to-provider ratios and community indicators of need, such as poverty or infant mortality, and are spatially designated by the Health Resources and Services Administration as geographic information system (GIS)–compatible shapefiles [14-16]. However, no comparable structure exists for the rehabilitation workforce, limiting visibility into how rehabilitation provider availability aligns with population need for rehabilitation services.

Existing workforce data sources, such as state licensure registries, are often reported only at the county level and rarely integrate demographic or health indicators [17-19]. These limitations obscure local variation and hinder efforts to identify areas of unmet need. Recent workforce projections in PT have highlighted the lack of precise practice location and individual-level demographic data as a limitation in modeling physical therapist supply and distribution [20]. GISs provide a powerful tool to evaluate health care access by combining location-based data with population characteristics and social indicators [21-24].

Recent GIS-based workforce studies across diverse health professions demonstrate the value of spatial methods for identifying disparities in provider availability. National analyses in the United States have revealed substantial geographic variation in accessibility to health care providers and dental clinics, including the emergence of “clinic deserts” in low-capacity regions [25,26]. Internationally, GIS-based assessments in Taiwan, Brazil, China, and Ethiopia have documented maldistribution of rehabilitation and primary care resources, highlighting clusters of poor accessibility and mismatches between service capacity and population need [27-31]. Together, these studies illustrate that workforce inequities often reflect fine-grained spatial patterns rather than simple urban-rural divides, underscoring the importance of small-area analysis. However, few studies have applied these methods to the rehabilitation workforce in the United States or used licensure data to generate tract-level availability estimates, leaving a notable gap that this study addressed.

Prior studies have used GISs to assess rehabilitation access in urban and rural contexts, revealing disparities in proximity to care, travel time, and service availability in relation to population health needs [32-36]. However, these studies have typically focused on either small metropolitan areas or broad, aggregated regions and have not offered standardized, reproducible methods for data collection, integration, and analysis at a smaller geographic scale. There is a growing need for research that documents transparent, scalable approaches to workforce mapping in rehabilitation. At present, no census tract–level GIS mapping exists for the rehabilitation workforce, leaving a gap in understanding how rehabilitation provider availability aligns with population need. Census tracts, which are smaller statistical subdivisions averaging 4000 residents, offer a valuable unit of analysis for this type of inquiry [37,38]. This feasibility study addressed that gap by developing and testing a transparent, replicable analytic approach for rehabilitation workforce mapping.

The objective of this study was to describe the feasibility of a GIS-based methodology to assess the availability of rehabilitation providers at the census tract level. This methodology systematically integrates state licensure data with open access demographic datasets to geocode provider locations and links them to area-level indicators of population need. The resulting spatial framework enables calculation of population-to-provider ratios, visualization of workforce distribution, and identification of small-area disparities in provider availability. By outlining each step, from data acquisition and cleaning to spatial analysis and mapping, this feasibility study is designed to support future research, workforce planning, and equity-focused policy development in rehabilitation.

## Methods

### Study Design

This was a descriptive, cross-sectional observational study designed to evaluate the availability of rehabilitation providers across census tracts using GISs. The primary objective was to estimate and compare population-to-provider ratios at the census tract level and examine how these ratios relate to the sociodemographic characteristics of each tract. This study used a methodology that combined state licensure data (obtained via paid public record requests) with open access demographic datasets. These data sources were integrated using spatial mapping techniques to facilitate small-area workforce analysis. Texas was chosen as the exemplar state because of its demographic diversity; pronounced urban-rural differences; and availability of comprehensive, high-quality licensure data. All processed datasets were stored locally in both CSV and geodatabase (.gdb) formats in ArcGIS Pro (Esri) to support spatial analysis.

When conducting GIS-based analyses of workforce availability, researchers should consider the geographic and demographic context of the region under study. Factors such as geographic land area, population density, and urban-rural distribution shape both the delivery and interpretation of health services and provider access.

Aggregated geographic units such as counties or zip codes, while commonly used, can mask meaningful local variation in provider availability and introduce misclassification bias. Zip codes, for example, often group together heterogeneous populations by combining areas of affluence with areas of poverty, which can distort analyses of access and need [39]. In contrast, census tracts, which typically include populations of 1200 to 8000 people and are designed to be socioeconomically homogeneous, provide a more precise basis for equitable spatial analysis [38].

The use of small-area units such as census tracts requires detailed data on provider location and population characteristics. This study’s methodology is designed to support census tract–level analysis when such data are available. Ultimately, GIS-based access assessments must balance granularity and feasibility to generate meaningful, context-sensitive insights.

### Ethical Considerations

This study methodology used secondary, deidentified data from publicly available sources, including licensure databases and census-based demographic datasets. No identifiable confidential information was disclosed, and no direct interaction with human subjects took place. Before implementation, this study was approved by the Texas Woman’s University Institutional Review Board as exempt under title 45 of the Code of Federal Regulations part 46.104(d) as the research involved publicly available or nonidentifiable data. Institutional review board approval was obtained before conducting data analysis. All data were deidentified before data cleaning and analysis. The institutional review approval is available in Multimedia Appendix 1.

### Data Sources

This study integrated information from two primary data sources: (1) state-level licensure data for rehabilitation professionals and (2) population and demographic data from the US Census Bureau’s American Community Survey (ACS). For this study, we used Texas as the example state in the analysis.

Licensure data were obtained from the Executive Council of Physical Therapy and Occupational Therapy Examiners through publicly available open record requests in Texas in 2022 [40]. This dataset included rehabilitation provider information, including residential mailing address (including street, city, state, and zip code); license type; issue and expiration dates; and basic demographic characteristics such as gender and ethnicity, where available. Personally identifiable information (eg, name and license number) were removed before analysis. Rehabilitation provider addresses were used for spatial analysis, and all data were stored and processed in a deidentified format. Although the licensure dataset included an optional work or practice address field, this information was often incomplete or inaccurate as many licensees either repeated their residential address or left the field blank. Therefore, residential mailing addresses were used as a consistent and complete proxy for spatial analysis.

Demographic and population data were sourced from the US Census Bureau’s 2020 ACS [41], a publicly available dataset providing census tract–level estimates for population size and characteristics such as race, ethnicity, age, disability status, insurance coverage, and income. Population estimates were obtained from the 2020 ACS dataset to match the licensure data time frame and provide reliable census tract–level denominators for ratio calculations. These data were downloaded as geodatabase files for integration with GIS software (eg, ArcGIS Pro).

These 2 data sources were used to estimate population-to-provider ratios and assess workforce availability at the census tract level.

### Data Aggregation and Cleaning

After obtaining licensure and demographic data, a multistep cleaning and aggregation process was conducted to ensure accurate spatial representation of the rehabilitation workforce.

#### Licensure Data Cleaning and Inclusion Criteria

Licensure datasets typically include all actively licensed rehabilitation professionals within a state or jurisdiction, including individuals practicing via interstate licensure compacts and those residing out of state. To ensure an accurate geographic representation of rehabilitation providers residing and working within the region of interest (Texas) the dataset was filtered based on the following criteria: (1) inclusion of individuals with an in-state residential address; (2) exclusion of licensees reporting only out-of-state addresses or inactive or retired licensure status; (3) retention of individuals with in-state residential addresses but missing workplace address data, with acknowledgment of potential uncertainty regarding employment status; and (4) manual review and correction of ambiguous or mislabeled geographic entries, such as addresses geocoded incorrectly or entries containing missing street-level information.

**Figure 1:**
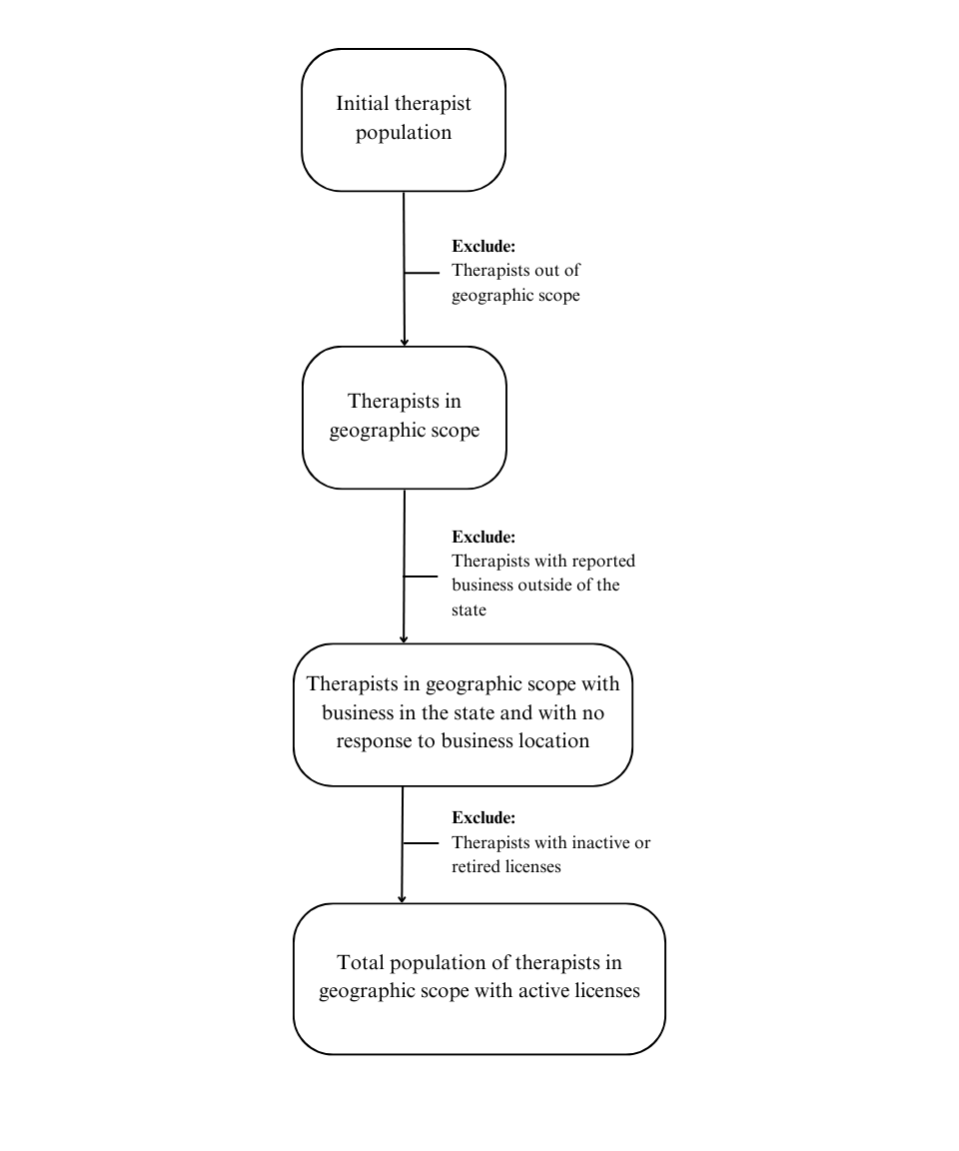
Licensure data cleaning process.

Figure 1 illustrates the selection and cleaning process, including inclusion and exclusion steps for licensure data. Filtering and cleaning procedures were conducted using R (version 4.3.1 or higher; R Foundation for Statistical Computing) within RStudio (Posit PBC). The code used for data cleaning is documented and available in Multimedia Appendices 2 and 3.

#### Software

All geospatial procedures were conducted using ArcGIS Pro (version 3.3), a GIS platform that allows for spatial data visualization, management, and analysis. Data cleaning and preprocessing were completed in RStudio before import into ArcGIS.

#### Demographic and Census Data Cleaning

Census tract–level data from the ACS were filtered to match the relevant geographic units used for licensure data aggregation. Relevant demographic variables (eg, population size, poverty rate, disability prevalence, and insurance coverage) were cleaned and formatted to allow for merging with provider datasets using census tract–level geographic identifiers.

### Geospatial Analysis Methods

#### Overview

In ArcGIS, for the purpose of visualization and analysis, the first step is to create a feature layer, which is a collection of similar geographic features that are grouped together [42]. Thus, feature layers were created for individual PT and OT datasets. These were combined into 1 feature layer with both PT and OT data that were used for analysis. Next, an ArcGIS geoprocessing tool called a spatial join that joins attributes from one feature to another based on the spatial relationship was added for data management. This spatial join was conducted for the combined PT and OT feature layer to match each rehabilitation provider spatially to the census tract in which their residential address was located. The join count resulting from this spatial join represents the number of providers residing in each of the 6896 census tracts from the 2020 census. This dataset was then merged with 2020 census tracts that included over 70 ACS demographic variables [43]. The full code for geocoding and spatial joins is included in Multimedia Appendix 4.

#### Calculating the Population-to-Provider Ratio

To estimate the population-to-provider ratio, a basic calculation of demand over supply was used. The population in the census tract, or the measure of the demand for services (*d_i_*), was divided by the provider join count, or the number of physical and occupational therapists residing in the census tract (*p_i_*):


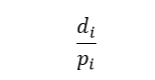


Additional adjustments were made to address zero-population and zero-provider tracts. Census tracts with zero population were excluded from ratio calculations as no residents indicate no potential demand for services. Because the population-to-provider ratio includes provider count in the denominator, tracts with zero providers would otherwise yield undefined values. To retain these tracts, which represent communities with residents but no local providers, a continuity correction was applied by substituting a denominator value of 1 for tracts with residents but zero providers. This approach preserved meaningful variation across all populated tracts while preventing division-by-zero and maintaining comparability across the dataset.

Although this method slightly attenuates extreme ratio values, it allows for visualization and analysis of areas where service absence is a critical feature of access inequity. This correction was selected over data exclusion or imputation to preserve geographic completeness and interpretability of spatial clustering. All adjustments were implemented in ArcGIS using a Python code block (Python Software Foundation), and the code is provided in Multimedia Appendix 4.

Next, using the adjusted provider count, the population-to-provider ratio was calculated by creating a new field and calculating that field by dividing the total population per census tract by the adjusted join count.

### Descriptive Mapping

The population-to-provider ratios across census tracts were investigated using descriptive statistics and choropleth and bivariate maps. A choropleth map is a thematic map in which areas are styled to represent the variation in a single variable. A bivariate map displays 2 variables using variations in colors or symbols, where each variable is assigned a different graded color scheme in a 3 × 3 table that is included in the top right-hand corner of the bivariate map [44,45]. A graduated color choropleth map was created to visually analyze the distribution of the population-to-provider ratios across the state of Texas by quintiles. Population-to-provider ratios were calculated and categorized into quintiles to facilitate relative comparison across areas following the approach used by Brown et al [46], who used quintile-based classifications to highlight spatial variation in the absence of established adequacy benchmarks. Comparing the highest and lowest quintiles facilitates the examination of factors associated with substantial difference in provider availability across areas. Bivariate choropleth maps were then created to visually describe the relationship between population-to-provider ratios and the percentage of disability in each census tract as defined by the ACS. Bivariate choropleth maps allow for the visualization of the specific areas that may be most in need of providers based on specific population demographics.

### Statistical Analyses

Descriptive statistics were used to describe the variability in population-to-provider ratio across all census tracts within the state of Texas. To describe differences in provider availability by rurality, census tracts were classified using 2020 Rural-Urban Commuting Area (RUCA) codes [47]. Tracts with a primary RUCA code of <4 were categorized as urban, and those with codes of ≥4 were categorized as rural. Each tract’s population-to-provider ratio was summarized by rurality using measures of central tendency and dispersion (median, IQR, minimum, and maximum). All analyses were conducted in R (version 4.3) using the *dplyr* and *ggplot2* packages. The R code for the RUCA analysis is included in Multimedia Appendix 5.

To evaluate whether the spatial distribution of access to rehabilitation providers exhibited clustering across the study area, a global Moran *I* statistic was computed for the population-to-provider ratio at the census tract level using ArcGIS Pro (version 3.3). Because Texas census tracts are polygonal units that vary substantially in geographic size, with very large tracts in rural regions and very small tracts in dense urban areas, a contiguity-based conceptualization of spatial relationships (edges only) was selected instead of a fixed distance threshold. This approach defines neighbors as tracts sharing a common boundary segment, ensuring that all tracts, regardless of their physical size, are evaluated based on direct adjacency rather than distance alone. Row standardization was applied to account for variability in the number of neighbors across tracts. The analysis tested whether the distribution of the population-to-provider ratio was spatially random, clustered, or dispersed using 999 random permutations to assess significance. *Z* scores and *P* values were used to evaluate departures from spatial randomness.

### Measures of Feasibility

The following measures of feasibility were assessed throughout the study: ability to obtain licensure data and the percentage of rehabilitation providers included after cleaning, the time and resources needed to clean and geocode the data, the percentage of provider addresses that were successfully geocoded, the ability to merge licensure data with ACS data, the percentage of census tracts that were matched, the feasibility of the geospatial analysis methods, and the relevance of the results to workforce planning and their replication.

## Results

### Overview

**Figure 2:**
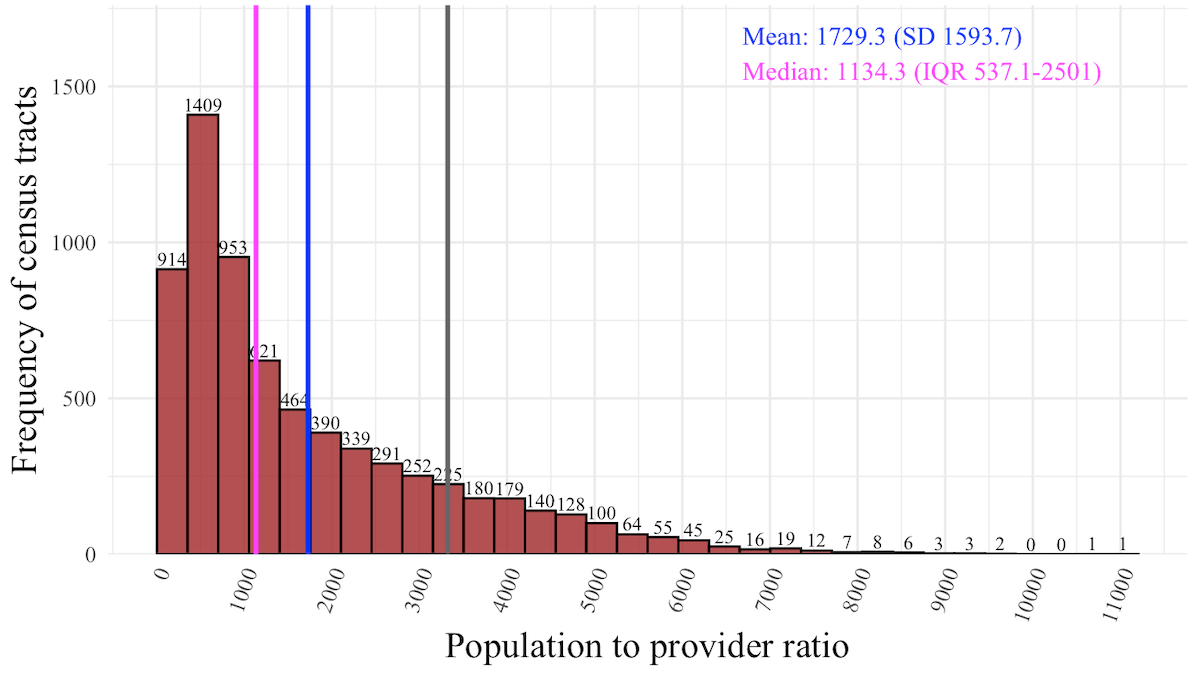
Distribution of population-to-provider ratios for rehabilitation providers.

Across 6896 tracts, ratios ranged from 4.5 to 11,147 persons per rehabilitation provider (median 1131, IQR 537-2501). Tracts with the lowest ratios clustered in the Texas Medical Center (eg, census tract 313102), whereas the highest ratios were observed in Lubbock (002100) and east of Dallas (census tract 012000). The distribution of population-to-provider ratios across the state of Texas is shown in Figure 2.

**Figure 3:**
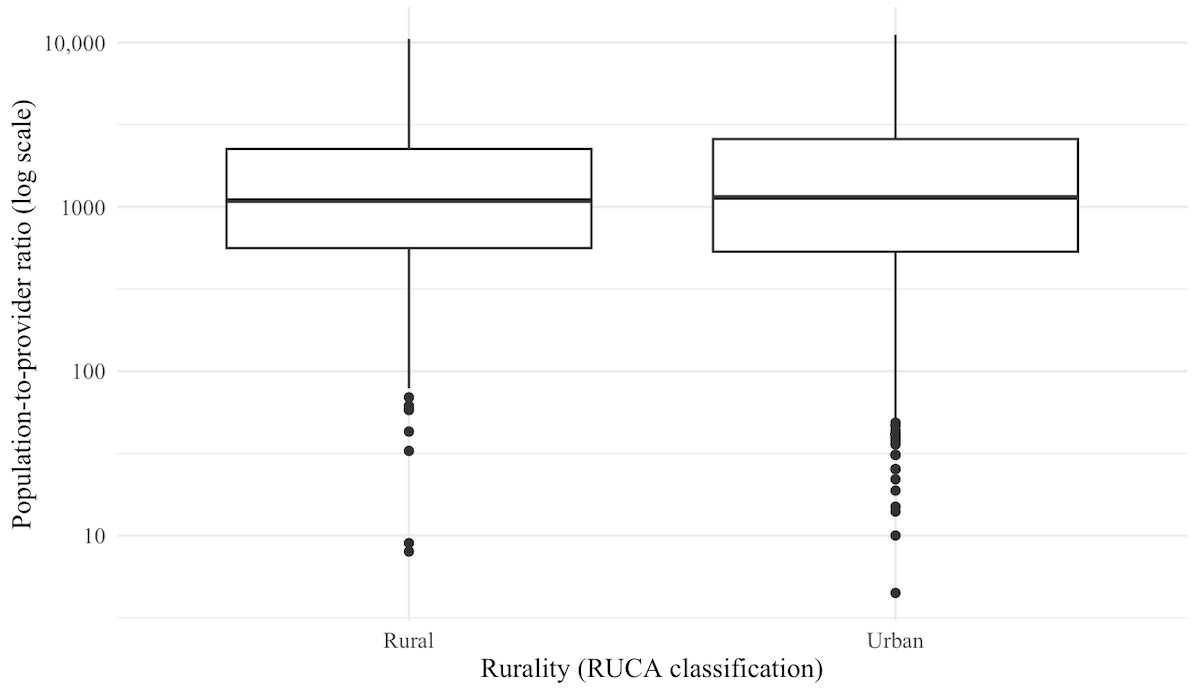
Distribution of population-to-provider ratios fro rehabilitation providers by rurality. RUCA: Rural-Urban Commuting Area.

When stratified by rurality, urban tracts (5734/6896, 83.1%) had a median ratio of 1141 (IQR 2054), whereas rural tracts (1162/6896, 16.9%) had a median ratio of 1093 (IQR 1690). These values indicate broadly similar distributions of provider availability between urban and rural areas, with slightly greater variability in urban tracts. Box plots (Figure 3) show the distribution of census tract–level population-to-provider ratios for rehabilitation providers classified by RUCA code. Ratios are displayed on a logarithmic scale to account for skewness in the data. Median ratios and IQRs are similar across rural and urban tracts, suggesting comparable variability in provider availability across both settings.

The global Moran *I* statistic for the population-to-provider ratio across census tracts was 0.305 (*Z*=40.28; *P*<.001), indicating significant positive spatial autocorrelation and less than a 1% chance that this clustered pattern was a result of random chance. The ArcGIS Moran *I* results are included in Multimedia Appendix 6.

**Figure 4:**
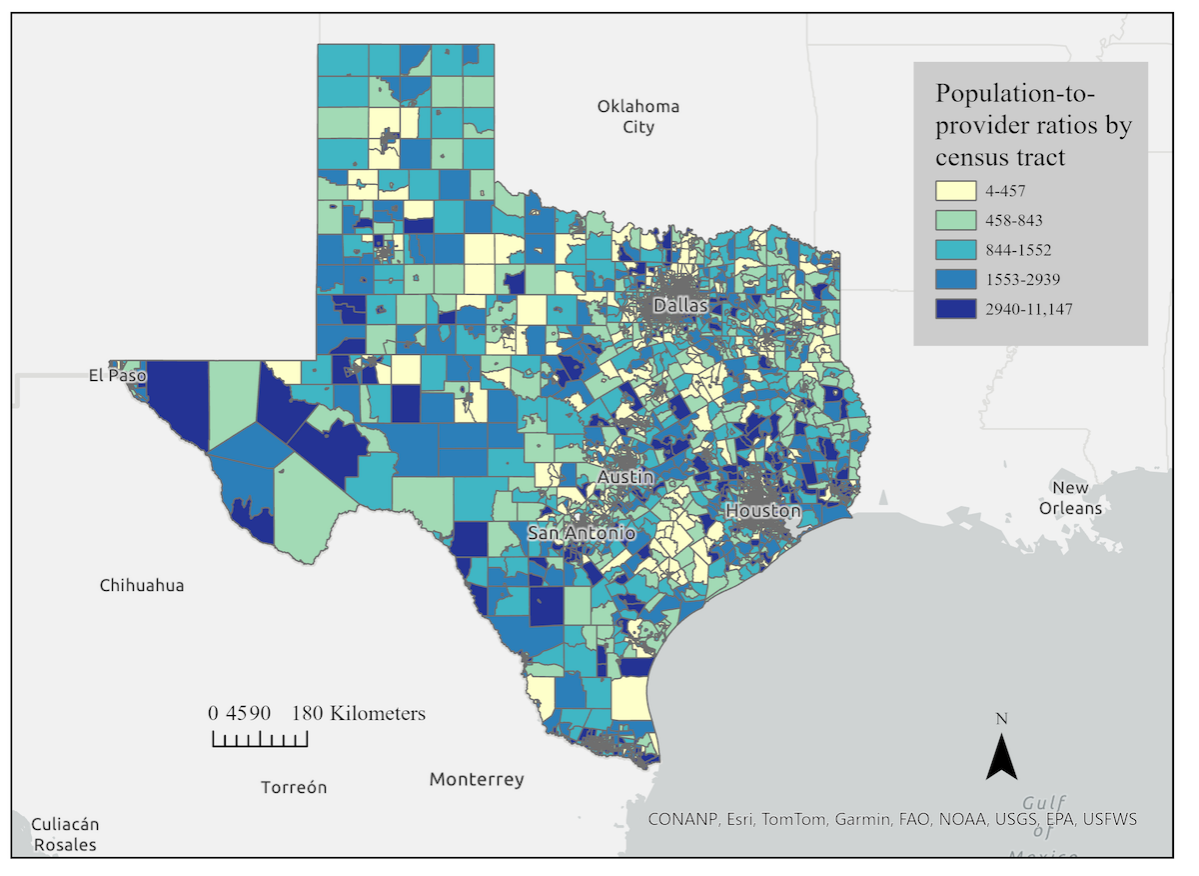
Spatial distribution of population-to-provider ratios for rehabilitation providers across Texas census tracts (combined occupational therapy and physical therapy providers).

Graduated and bivariate choropleth maps were used to visualize provider availability and its intersection with social and demographic indicators. A graduated color choropleth map (Figure 4) displays population-to-provider ratios across all census tracts in Texas, with lighter shades indicating higher provider availability (lower population-to-provider ratio) and darker shades indicating lower provider availability.

To further illustrate equity-related patterns, bivariate choropleth maps overlay these ratios with variables such as percentage of disability, percentage of minority populations, and percentage of the population below the poverty level. For example, disability data were drawn from 6 ACS questions addressing hearing, vision, cognitive ability, ambulatory ability, self-care, and independent living difficulty [48]; respondents answering “yes” to any item were classified as having a disability.

**Figure 5:**
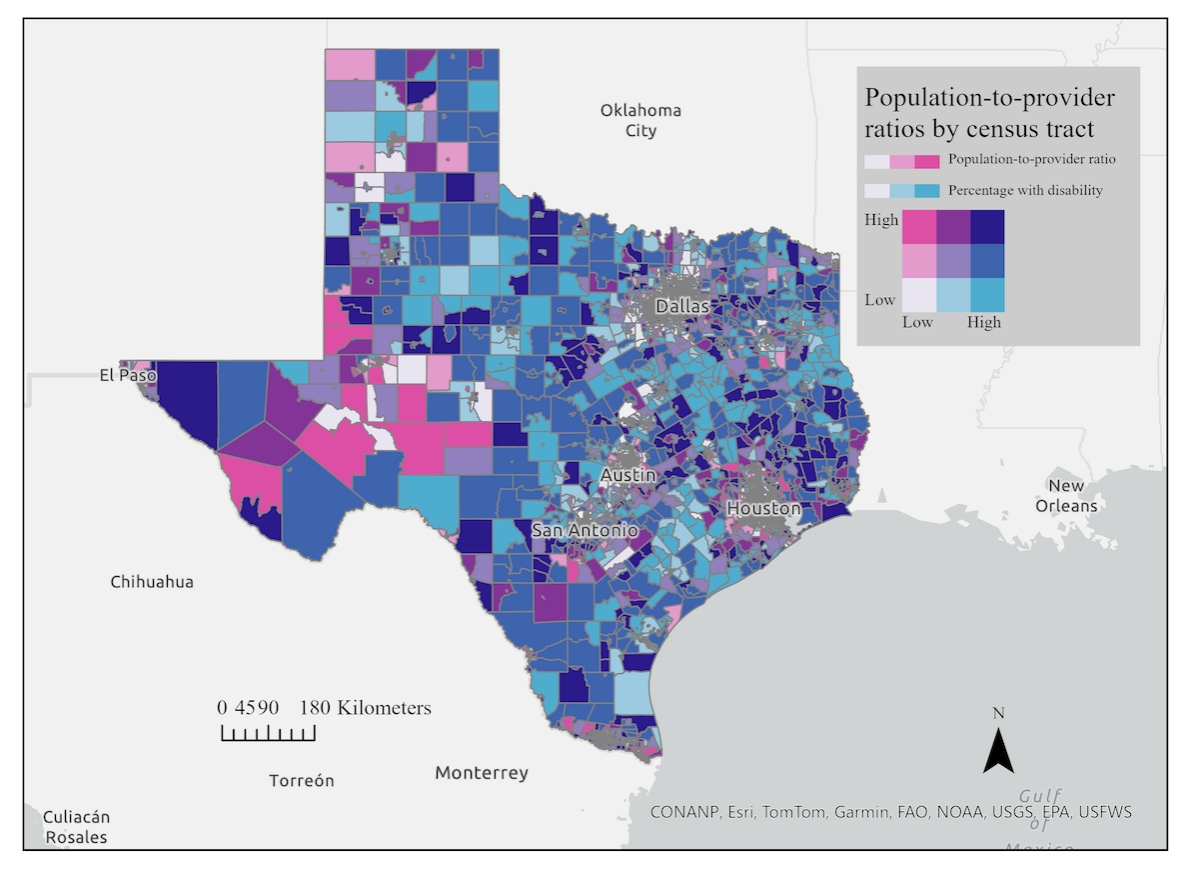
Bivariate choropleth map by quintile of the relationship between population-to-provider ratio (for rehabilitation providers) and percentage of disability within each census tract in Texas.

As shown in Figure 5, the overlay of population-to-provider ratios with disability prevalence reveals regional variability across the state, although not a strict urban-rural divide. While areas of west and south Texas include clusters of high population-to-provider ratios, similar patterns also appear in parts of urban and periurban counties. Conversely, some rural tracts demonstrate moderate ratios despite small provider counts, reflecting the influence of low population density on the calculation. Regions in dark blue reflect regions where there is a high population-to-provider ratio coinciding with high prevalence of disability, suggestive of census tracts where there is a mismatch of population need and provider availability.

### Feasibility

Feasibility was assessed throughout each phase of the methods, including data acquisition, data processing, descriptive mapping, and statistical analysis. Table 1 illustrates the measures of feasibility assessed in this study.

**Table 1:** Feasibility results.

Domain	Indicator	Results
Data acquisition	Ability to obtain licensure data; percentage of licensed rehabilitation providers included after cleaning	Licensure data were purchased and accessible in 2022; 28,613 PTs^a^ (88% of initial entries) and 16,501 OTs^b^ (87% of initial entries) were retained after cleaning.
Data processing	Time and resources needed to clean and geocode data	Data cleaning required manual review of address inconsistencies and development of flowcharts and R code. Processing took approximately 4 weeks of graduate assistant time with regular oversight by the investigators.
Geocoding success	Percentage of rehabilitation provider addresses successfully geocoded	Nearly all addresses were successfully geocoded; only 9 PT and 5 OT addresses (<0.05% of total entries) were excluded because they could not be verified.
Data integration	Ability to merge licensure data with ACS^c^ data; percentage of tracts matched	Rehabilitation provider data (28,613 PTs and 16,501 OTs) were successfully matched to 6896 Texas census tracts and merged with ≥70 ACS variables, with no unmatched tracts.
Analysis of feasibility	Ability to generate population-to-provider ratios, maps, and bivariate overlays	Ratios were calculated across all tracts; maps and overlays were successfully created using ArcGIS. Analyses were replicable using shared R code and ArcGIS workflows.
Acceptability and utility	Relevance for workforce planning; potential for replication	Methods allowed for visualization of geographic disparities in provider availability. The workflows (R scripts, flow diagrams, and verification steps) are reproducible and adaptable to other states and health professions, although feasibility will depend on state-specific licensure access rules.

^a^PT: physical therapist.

^b^OT: occupational therapist.

^c^ACS: American Community Survey.

## Discussion

### Principal Findings

Rehabilitation provider availability showed substantial geographic variation, with tract-level population-to-provider ratios ranging from 4.5 to 11,147 (median 1131, IQR 2010) and similar central tendencies across rural and urban areas, yet the strong positive spatial autocorrelation (Moran *I*=0.305; *P*<.001) indicated clear clustering of high- and low-availability regions.

These patterns align with findings from other GIS-based workforce studies across a range of health professions, which similarly report uneven spatial distribution of health care providers and localized pockets of limited access. National analyses in the United States have identified substantial variation in spatial accessibility to health care providers and dental clinics [25,26], whereas international assessments in Taiwan, Brazil, China, and Ethiopia have documented comparable clustering of high- and low-access areas across rehabilitation and community care services [27-31]. Although these studies differ in setting and methodological approach, they consistently demonstrate that workforce maldistribution emerges at small geographic scales and often reflects underlying sociodemographic gradients. This study extends this body of work to the US rehabilitation workforce by applying comparable geospatial methods to tract-level licensure data, situating our findings within a broader pattern of spatially concentrated workforce inequities.

Statewide patterns in this study demonstrated striking variation in population-to-provider ratios across census tracts, revealing local disparities that county-level summaries fail to capture. Previous statewide reports from the Texas Health Professions Resource Center have suggested that availability challenges are largely a rural issue [17,18]. In 2022, the average population-to-provider ratio for physical therapists was 1593:1 in metropolitan counties and 2870:1 in nonmetropolitan counties, whereas for occupational therapists, it was 2778:1 in metropolitan areas and 5611:1 in nonmetropolitan areas. These data painted a picture of a binary divide between urban and rural access.

When we examined rehabilitation provider availability at the census tract level rather than the more common county designation, our findings diverged from prevailing assumptions. Although state-level reports show markedly higher population-to-provider ratios in nonmetropolitan counties than in metropolitan ones, our tract-level analysis found similar median values for urban and rural tracts, albeit with greater variability in urban areas. This pattern mirrors findings for other health professions: for example, a spatial analysis of primary care access in Philadelphia, Pennsylvania, revealed that geographic access disparities often overlay racial and socioeconomic patterns rather than simply urban versus rural gradients [46]. By shifting to smaller geographic units, our study revealed that within-urban and within-rural heterogeneity matters because access gaps may persist in urban neighborhoods that are often assumed to be well served and, conversely, some rural tracts may fare better than county-level data suggest.

The Moran *I* statistic confirms that rehabilitation provider availability is spatially clustered rather than randomly distributed. In other words, tracts with low provider availability tend to be near one another, forming regional patterns of limited access. A comparable spatial analysis of rehabilitation human resources in China demonstrated this clustering effect across provinces, underscoring that workforce maldistribution may follow geographic spillovers rather than isolated pockets [49]. However, in our Texas analysis, these clusters did not correspond neatly to urban or rural classifications, indicating that factors beyond simple rurality may drive spatial patterns of provider scarcity. Although further research should investigate these determinants in depth, the observed clustering suggests that improving access will likely require coordinated, regionally targeted interventions rather than isolated, tract-by-tract fixes.

Although this analysis was based on provider residential addresses, the presence of strong spatial clustering indicates that these data still meaningfully represent patterns of service availability. It is unlikely that large numbers of providers are commuting long distances into the clusters of low access identified in this study. Instead, these regions likely represent genuine shortages in the local rehabilitation workforce. Recognizing and addressing these gaps is essential not only from an equity standpoint but also from a broader population health perspective. Limited access to rehabilitation care can lead to preventable disability, loss of function, and reduced participation in work and community life [50,51].

The GIS-based maps generated in this study extend beyond visualization: they function as actionable tools for workforce assessment and planning. Similar spatial approaches have been applied in other areas of public health to identify regions where need and provider availability intersect, informing policy and resource allocation decisions. For example, bivariate choropleth maps have been used in cancer control to guide the placement of screening resources in areas with low service capacity and high disease burden [52] and in studies of lung cancer screening to identify US counties with both high smoking prevalence and low provider density [53]. Such mapping frameworks demonstrate the potential of GIS-based methods to translate complex population and workforce data into actionable insights. Applying this approach to rehabilitation introduces an equivalent capacity to identify where population need and provider scarcity converge. By pinpointing census tracts with high disability prevalence and low provider availability, these maps enable decision-makers to prioritize areas for resource allocation, community partnerships, and program expansion. Beyond workforce planning, they also provide a framework for longitudinal monitoring, allowing policymakers to observe how workforce distribution shifts in response to educational pipelines, policy incentives, or population change over time. In this way, the maps produced in this study serve not only as evidence of current inequities but also as instruments for promoting future workforce equity in rehabilitation.

### Strengths and Limitations

The approach presented in this paper is replicable; the step-by-step procedures, along with the inclusion of code in the multimedia appendices, are intentional so that the methods can be applied in a variety of geographic contexts or across different health professions. This transparency supports reproducibility and scalability in future research. Additionally, the novel method emphasizes high geographic resolution by conducting analyses at the census tract level. This finer scale allows for a more precise identification of geographic disparities than approaches that rely on broader units such as counties or zip codes [38,39], offering a more detailed understanding of provider availability.

There are several limitations to this study. First, it focused exclusively on availability, one domain within the theory of access by Penchansky and Thomas [7], and did not account for realized access (the actual entry of a patient into the health care system) [54] or outcomes. The use of residential addresses as a proxy for provider practice location introduced potential misclassification, and the population-to-provider ratio did not capture factors such as transportation infrastructure, health insurance coverage, or service capacity.

Additionally, the observational nature of the analysis precludes causal inferences about relationships between availability and health disparities. This study also prioritized comparison of high- and low-availability areas using quintiles, which may not account for regional variations in land mass, population density, or local health systems.

Finally, publicly available licensure and census data are subject to reporting errors and definitional inconsistencies. These data limitations should be acknowledged in future research and addressed, where possible, through improved workforce data infrastructure.

### Future Applications

The methods used in this study are adaptable and scalable for use in other professions (eg, speech-language pathology and behavioral health) and other geographic regions. Future studies can build on this framework by incorporating clinic location data, travel time analyses, and patient-level outcomes to provide a more complete picture of access to care. Integration of GISs into national workforce planning tools could support more equitable distribution of health services and guide data-driven decisions at local, state, and federal levels.

Beyond immediate replication, this work lays the foundation for a broader research agenda in rehabilitation workforce equity. The GIS-based approach demonstrated in this paper establishes a platform for developing rehabilitation-specific shortage area indexes, evaluating spatial alignment between workforce supply and population need, and examining equity in workforce diversity. These next steps extend beyond feasibility to inform national policy and education initiatives aimed at improving access for underserved communities. The ability to acquire, clean, and link licensure and census data demonstrates both the practicality and potential of applying this method to larger, longitudinal studies that track changes in provider availability and access over time. In this way, the results directly inform workforce planning by providing an evidence-based foundation for policies and programs aimed at achieving a more equitable distribution of rehabilitation providers.

### Conclusions

This study demonstrates the feasibility and value of integrating licensure and demographic data to assess rehabilitation provider availability at the census tract level, which is a novel approach in this field. Quantitatively, tract-level population-to-provider ratios ranged from 4.5 to 11,147 (median 1131, IQR 2010) and demonstrated significant spatial clustering (Moran *I*=0.305; *P*<.001). The results reveal that provider availability in Texas is spatially clustered and influenced by factors beyond urban-rural status, highlighting the need for targeted, regionally informed interventions.

By establishing a replicable, GIS-based framework, this work advances both workforce equity research and geospatial health methods, offering a foundation for small-area analyses that move beyond traditional county-level approaches. These findings underscore the potential of spatial mapping to identify underserved communities and guide data-driven workforce planning and policy development that promote equitable access to rehabilitation care.

## Appendix

Multimedia Appendix 1 Institutional review board approval letter from Texas Woman’s University for this research protocol.

Multimedia Appendix 2 R code for cleaning physical therapist licensure data, creating subsets for spatial and statistical analysis.

Multimedia Appendix 3 R code for cleaning occupational therapist licensure data, creating subsets for spatial and statistical analysis.

Multimedia Appendix 4 Python code block for ArcGIS reclassifications.

Multimedia Appendix 5 R code for Rural-Urban Commuting Area classification analysis.

Multimedia Appendix 6 Moran I ArcGIS results output.

## Abbreviations

ACS: American Community Survey
GIS: geographic information system
OT: occupational therapy
PT: physical therapy
RUCA: Rural-Urban Commuting Area

## Data Availability

The datasets generated or analyzed during this study are available from the corresponding author on reasonable request.
