# Supplementary material for: Mapping the Availability of Rehabilitation Providers Using Public Licensure and Population Data for a Geographic Information System–Based Approach to Workforce Planning: Cross-Sectional Feasibility Study
Source: JMIR Form Res. 2025 Dec 23;9:e85025. doi: 10.2196/85025 (PMC12775756; doi:10.2196/85025)
Supplement: Multimedia Appendix 1 [file formative_v9i1e85025_app1.pdf]

TWU

Madeline Ratoza <mratoza@twu.edu>

IRB-FY2023-162 - Renewal (Extension) Letter

1 message

do-not-reply@cayuse.com <do-not-reply@cayuse.com> Thu, Mar 20, 2025 at 11:14 AM
To: kmitchell@twu.edu, mratoza@twu.edu, rpatel@twu.edu, wbrewer@twu.edu

Texas Woman's University
Institutional Review Board (IRB)
irb@twu.edu
https://www.twu.edu/institutional-review-board-irb/

March 20, 2025

Madeline Ratoza
Physical Therapy - Houston, Physical Therapy - Denton

Re: Renewal - IRB-FY2023-162 Modeling Access in Rehabilitation Using Population Health Data

Dear Madeline Ratoza,

The renewal for the above referenced study has been reviewed and approved on March 20, 2025 by TWU IRB - Houston.

Note that any modifications to this study must be submitted for IRB review prior to their implementation, including the submission of any agency approval letters, changes in research personnel, and any changes in study procedures or instruments. Additionally, the IRB must be notified immediately of any adverse events or unanticipated problems. All modification requests, incident reports, and requests to close the file must be submitted through Cayuse.

Approval for this study will now expire on March 19, 2026. A reminder of the study expiration will be sent 45 days prior to the expiration. If the study is ongoing, you will be required to submit a renewal request. When the study is complete, a close request may be submitted to close the study file.

If you have any questions or need additional information, please email your IRB analyst at irb@twu.edu or refer to the IRB website.

Sincerely,

TWU IRB - Houston
