# Supplementary material for: Mapping the Availability of Rehabilitation Providers Using Public Licensure and Population Data for a Geographic Information System–Based Approach to Workforce Planning: Cross-Sectional Feasibility Study
Source: JMIR Form Res. 2025 Dec 23;9:e85025. doi: 10.2196/85025 (PMC12775756; doi:10.2196/85025)
Supplement: Multimedia Appendix 2 [file formative_v9i1e85025_app2.pdf]

```r
################################################################
# Title: Data Cleaning for Texas Physical Therapist Licensure Data
# Author: Madeline Ratoza
# Purpose:
#   - Load PT licensure data from Excel
#   - Check categorical values
#   - Recode business state fields
#   - Identify providers living vs working in Texas
#   - Summarize counts of rows by state combinations
#   - Create subsets for spatial analysis
# Input:
#   - PT_Real.xlsx
# Output:
#   - Cleaned dataframes for mapping and statistical analysis
# Packages:
#   - readxl
#   - stringr
#   - dplyr
################################################################

# library
library(readxl) # import excel
library(stringr) # string replace
library(dplyr) # used for mutate

# Import data
PT_Real <- read_excel("PT_Real.xlsx")

# Check categorical variables
table(PT_Real$State)
table(PT_Real$BusinessState)

# Replace “T” with “TX” and “TC” with “TX”
PT_Real <- PT_Real %>% mutate(BusinessState1 = case_when(
  BusinessState == "T" ~ "TX",
  BusinessState == "TC" ~ "TX",
  TRUE ~ BusinessState))

# Table to show frequency of business state with the old and new Business state variable
table(PT_Real$BusinessState)
table(PT_Real$BusinessState1)

# Working in TX but living in another state
PT_Real <- PT_Real %>% mutate(W_TX = if_else(BusinessState == "TX" & State != "TX", 1, 0))

# Table showing those working in TX but living in another state
table(PT_Real$W_TX, PT_Real$BusinessState)
table(PT_Real$W_TX, PT_Real$State)

# Check variable names
nrow(PT_Real)
variable.names(PT_Real)

# Summaries
nrow(PT_Real)
sum(PT_Real$State == "TX", na.rm = TRUE)
sum(is.na(PT_Real$State))
sum(PT_Real$State != "TX", na.rm = TRUE)
sum(PT_Real$BusinessState == "TX", na.rm = TRUE)
sum(PT_Real$BusinessState != "TX", na.rm = TRUE)
sum(is.na(PT_Real$BusinessState))
nrow(PT_Real[PT_Real$State %in% "TX" & PT_Real$BusinessState %in% "TX", ])
```

```
nrow(PT_Real[PT_Real$State %in% "TX" & is.na(PT_Real$BusinessState), ])

# Create new datasets
new_dataPT <- subset(PT_Real, PT_Real$State == "TX")
new_dataPT2 <- subset(PT_Real, PT_Real$State == "TX" & PT_Real$BusinessState == "TX")
new_dataPT3 <- subset(new_dataPT, new_dataPT$BusinessState1 == "TX" |
is.na(new_dataPT$BusinessState))
new_dataPT4 <- subset(new_dataPT3, new_dataPT3$LicenseStatus == "Permanent" |
new_dataPT3$LicenseStatus == "Temporary SCP")
```
