# Supplementary material for: Mapping the Availability of Rehabilitation Providers Using Public Licensure and Population Data for a Geographic Information System–Based Approach to Workforce Planning: Cross-Sectional Feasibility Study
Source: JMIR Form Res. 2025 Dec 23;9:e85025. doi: 10.2196/85025 (PMC12775756; doi:10.2196/85025)
Supplement: Multimedia Appendix 3 [file formative_v9i1e85025_app3.pdf]

```
##############################################################
# Title: Data Cleaning for Texas Occupational Therapist Licensure Data
# Author: Madeline Ratoza
# Purpose:
#   - Load OT licensure data from Excel
#   - Check categorical values
#   - Identify providers living vs working in Texas
#   - Summarize counts of rows by state combinations
#   - Create subsets for spatial analysis
# Input:
#   - OT_Real.xlsx
# Output:
#   - Cleaned dataframes for mapping and statistical analysis
# Packages:
#   - readxl
#   - stringr
#   - dplyr
##############################################################

# library
library(readxl) # import excel
library(stringr) # string replace
library(dplyr) # used for mutate

# Import data
OT_Real <- read_excel("OT_Real.xlsx")

# Check categorical variables
table(OT_Real$State)
table(OT_Real$BusinessState)

# Working in TX but living in another state
OT_Real <- OT_Real %>% mutate(W_TX = if_else(BusinessState == "TX" & State != "TX", 1, 0))

# Table showing those working in TX but living in another state
table(OT_Real$W_TX, OT_Real$BusinessState)
table(OT_Real$W_TX, OT_Real$State)

# Check variable names
nrow(OT_Real)
variable.names(OT_Real)

# Summaries
nrow(OT_Real)
sum(OT_Real$State == "TX", na.rm = TRUE)
sum(is.na(OT_Real$State))
sum(OT_Real$State != "TX", na.rm = TRUE)
sum(OT_Real$BusinessState == "TX", na.rm = TRUE)
sum(OT_Real$BusinessState != "TX", na.rm = TRUE)
sum(is.na(OT_Real$BusinessState))
nrow(OT_Real[OT_Real$State %in% "TX" & OT_Real$BusinessState %in% "TX", ])
nrow(OT_Real[OT_Real$State %in% "TX" & is.na(OT_Real$BusinessState), ])

# Create new datasets
new_dataOT <- subset(OT_Real, OT_Real$State == "TX")
new_dataOT2 <- subset(OT_Real, OT_Real$State == "TX" & OT_Real$BusinessState == "TX")
new_dataOT3 <- subset(new_dataOT, new_dataOT$BusinessState == "TX" |
is.na(new_dataOT$BusinessState))
new_dataOT4 <- subset(new_dataOT3, !new_dataOT3$LicenseStatus %in% c("Retired",
"Inactive"))
```
