# Supplementary material for: Mapping the Availability of Rehabilitation Providers Using Public Licensure and Population Data for a Geographic Information System–Based Approach to Workforce Planning: Cross-Sectional Feasibility Study
Source: JMIR Form Res. 2025 Dec 23;9:e85025. doi: 10.2196/85025 (PMC12775756; doi:10.2196/85025)
Supplement: Multimedia Appendix 4 [file formative_v9i1e85025_app4.pdf]

```
##############################################################
# Title: ArcGIS Pro Python Code Block for Reclassify
# Author: Madeline Ratoza
# Purpose:
#   - Avoid divide-by-zero errors in provider ratio calculations
#   - Reclassify zero join counts to value of 1
# Input:
#   - BlockGroups_SpatialJoin3 feature class/table
# Output:
#   - Field with reclassified join counts
# Tool:
#   - arcpy.management.ReclassifyField
##############################################################

import arcpy

arcpy.management.ReclassifyField(
    in_table="BlockGroups_SpatialJoin3",
    field="Join_Count",
    expression="0",
    code_block="""
def reclass(val):
    if val == 0:
        return 1
    else:
        return val
""",
    data_type="LONG"
)
```
