# Supplementary material for: Mapping the Availability of Rehabilitation Providers Using Public Licensure and Population Data for a Geographic Information System–Based Approach to Workforce Planning: Cross-Sectional Feasibility Study
Source: JMIR Form Res. 2025 Dec 23;9:e85025. doi: 10.2196/85025 (PMC12775756; doi:10.2196/85025)
Supplement: Multimedia Appendix 5 [file formative_v9i1e85025_app5.pdf]

Urban-Rural Analysis

Madeline Ratoza

2025-11-10

```r
setwd("/Users/madelineratoza/Desktop")
getwd()
```

```
## [1] "/Users/madelineratoza/Desktop"
```

```r
list.files()
```

```
##   [1] "~$_Interview_3.docx"
##   [2] "~$23 Informatics Conference Abstract.docx"
##   [3] "~$ans in Limbo.docx"
##   [4] "~$C - Unit 9 Sync Class.docx"
##   [5] "~$C Draft.docx"
##   [6] "~$CAMPUS_Res 1 D Comp Exam_Fall25 (Duplicate).xlsx"
##   [7] "~$ex Curricular Map.docx"
##   [8] "~$FLSA .xlsx"
##   [9] "~$hedules.docx"
##  [10] "~$holarship Form Ratoza 2022.docx"
##  [11] "~$imbursment form ELC 2023 Ratoza.doc"
##  [12] "~$Item Writing Presentation .pptx"
##  [13] "~$Item Writing Presentation.pptx"
##  [14] "~$K I Lab Mock Evaluation Case 1 - Cervical Radiculopathy_MR.docx"
##  [15] "~$K I Lab Mock Evaluation Case 1 Medical Intake_MR.docx"
##  [16] "~$K I Lab Mock Evaluation Case 2 Medical Intake_MR.docx"
##  [17] "~$rgan Tracking Changes Contextual Information Lab Activity[48].docx"
##  [18] "~$TA CICP II ICE Cat 1 Curriculum Matrix_ICEIP.docx"
##  [19] "~$tside Employment Form 2019.doc"
##  [20] "~$ving the Way Forward.docx"
##  [21] "$RECYCLE.BIN"
##  [22] "001_Texas_Accessibility"
##  [23] "001_Texas_Availability"
##  [24] "001_Texas_RPSA"
##  [25] "002_Region1_MSARLA"
##  [26] "1.1 Data FINAL OT 16507 .xlsx"
##  [27] "1.1 Providers Combined.xlsx"
##  [28] "Acute.xlsx"
##  [29] "AoE Research Grant Application.docx"
##  [30] "ASSSMENT DOCUMENTS"
##  [31] "BPA.xlsx"
##  [32] "BPA2.xlsx"
##  [33] "Brown et al. 2016 - Health Aff_.pdf"
##  [34] "CASM M SP.xlsx"
##  [35] "Clinic Loation OP Only For GIS.csv"
##  [36] "Clinic Loation OP Only For GIS.xlsx"
```

```
## [37] "Comp Exam Data Analysis Fall 2025 Subterm D Term 1.xlsx"
## [38] "Comp Exam Data Analysis Fall 2025 Subterm D Term 2.xlsx"
## [39] "Comp Exam Data Analysis Fall 2025 Subterm D Term 3.xlsx"
## [40] "Comp Exam Data Analysis Fall 2025 Subterm D Term 4.xlsx"
## [41] "Comp Exam Data Analysis Flex Only EIP.xlsx"
## [42] "Comp Exam Data Analysis Template_MAKE A COPY_v2.xlsx"
## [43] "Comp Exam Dates (Draft) Spring 2025.xlsx"
## [44] "Course Director Feedback Subterm D Comprehensive Exam Fall 2025.xlsx"
## [45] "CR_C.xlsx"
## [46] "CR+C ALL EXAM QUESTIONS.pdf"
## [47] "CRC CASM Res.xlsx"
## [48] "Dallas Scores.xlsx"
## [49] "desktop.ini"
## [50] "EIP.xlsx"
## [51] "EIPA.xlsx"
## [52] "ES+P 11_5.pdf"
## [53] "Faculty Council Bylaws 10_22_25.docx"
## [54] "Faculty Council Meeting Organization.xlsx"
## [55] "FINAL STUDENT SPREADSHEETS"
## [56] "FLMI.xlsx"
## [57] "FLSA.xlsx"
## [58] "FundDallas.xlsx"
## [59] "Hotspotdata.xlsx"
## [60] "Hotspotlogreg.xlsx"
## [61] "HPSA1.xlsx"
## [62] "IFS Exam 2 feedback.xlsx"
## [63] "Jim.xlsx"
## [64] "MSK1 Flex CASM.xlsx"
## [65] "MSK3 FLSA Res.xlsx"
## [66] "NMI Flex TXAU.xlsx"
## [67] "NMIFLSARES.xlsx"
## [68] "NMIV11_7.pdf"
## [69] "OptimizedHS.xlsx"
## [70] "Podcast Things "
## [71] "Poptoprovider.xlsx"
## [72] "Presentation1.pptx"
## [73] "ProIntegratingData 2"
## [74] "Proposal for Special Appointment.docx"
## [75] "PSAC.xlsx"
## [76] "Ratio.xls"
## [77] "Redistricting Summer.Rmd"
## [78] "RPSA.xlsx"
## [79] "RPSA1.xlsx"
## [80] "RPSA4.xls"
## [81] "RPSA4HPSA.xls"
## [82] "RPSA4HPSA1.xls"
## [83] "RPSA4toPCHPSA.xlsx"
## [84] "RUCA-codes-2020-tract.xlsx"
## [85] "Screen Recording 2025-10-14 at 3.10.28 PM.mov"
## [86] "Screen Recording 2025-10-14 at 3.21.10 PM.mov"
## [87] "Screen Recording 2025-10-27 at 8.33.32 PM.mov"
## [88] "Screen Recording 2025-10-29 at 4.12.27 PM.mov"
## [89] "Screen Recording 2025-10-29 at 8.35.16 AM.mov"
## [90] "Screen Recording 2025-10-29 at 8.36.03 AM.mov"
```

```
##  [91] "Screen Recording 2025-10-29 at 8.36.25 AM.mov"
##  [92] "Screen Recording 2025-10-29 at 8.49.20 AM.mov"
##  [93] "Screenshot 2025-10-07 at 12.18.58 PM.png"
##  [94] "Screenshot 2025-10-07 at 12.22.18 PM.png"
##  [95] "Screenshot 2025-10-07 at 2.25.28 PM.png"
##  [96] "SHHS Spring Overview.xlsx"
##  [97] "SpatialRelsIntro"
##  [98] "StartAnalytics 2"
##  [99] "Student - Absence Approval Form-1.pdf"
## [100] "STUDENT_CATEGORY_PERFORMANCE.D10-29-25T18_38.U55b73c7e-6315-4c86-85ba-a765f201212b 2.xlsx"
## [101] "STUDENT_CATEGORY_PERFORMANCE.D10-30-25T11_06.xlsx"
## [102] "sudo port install git"
## [103] "SummarizeData"
## [104] "T_L Flex CASM.xlsx"
## [105] "T+L All Questions.pdf"
## [106] "T+L.xlsx"
## [107] "Texas Redistricting.Rmd"
## [108] "THE ONE.xls"
## [109] "Thumbs.db"
## [110] "To print Parker"
## [111] "WildernessAreas"
```

```r
# View the column names to verify
colnames(ratio_data)
```

```
## [1] "OBJECTID"                        "GEOID"
## [3] "State"                           "County"
## [5] "tract"                           "PopulationtoProviderZerotoOne"
## [7] "Quintile"
```

```r
# Summarize the Population-to-Provider Ratio variable
ratio_summary <- ratio_data %>%
  summarize(
    n = n(),
    min_value = min(PopulationtoProviderZerotoOne, na.rm = TRUE),
    q1 = quantile(PopulationtoProviderZerotoOne, 0.25, na.rm = TRUE),
    median_value = median(PopulationtoProviderZerotoOne, na.rm = TRUE),
    q3 = quantile(PopulationtoProviderZerotoOne, 0.75, na.rm = TRUE),
    max_value = max(PopulationtoProviderZerotoOne, na.rm = TRUE),
    mean_value = mean(PopulationtoProviderZerotoOne, na.rm = TRUE),
    sd_value = sd(PopulationtoProviderZerotoOne, na.rm = TRUE)
  )

print(ratio_summary)
```

```
## # A tibble: 1 x 8
##       n min_value    q1 median_value    q3 max_value mean_value sd_value
##   <int>     <dbl> <dbl>        <dbl> <dbl>     <dbl>      <dbl>    <dbl>
## 1  6896      4.48  537.        1134.  2501     11147      1729.    1594.
```

```r
hist(ratio_data$PopulationtoProviderZerotoOne,
     breaks = 40,
     main = "Distribution of Population-to-Provider Ratios",
     xlab = "Population-to-Provider Ratio",
     col = "lightblue")
```

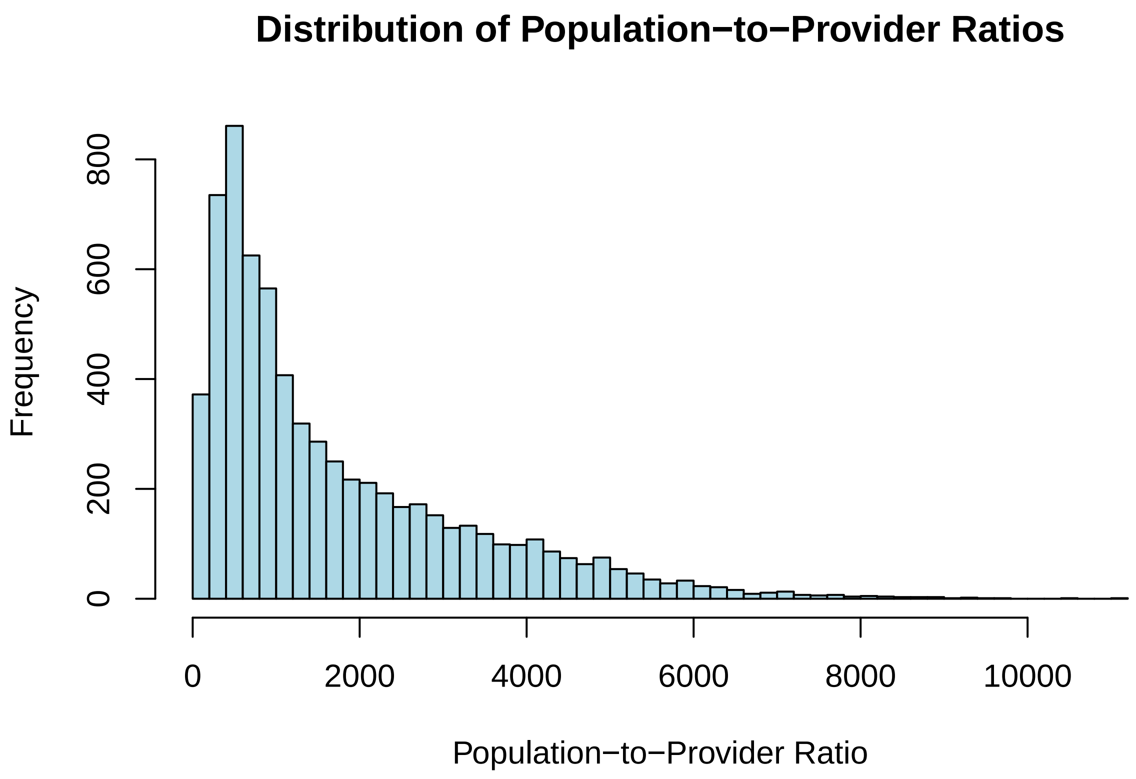


```r
# Step 1: List all sheets to see what's inside
excel_sheets("/Users/madelineratoza/Desktop/RUCA-codes-2020-tract.xlsx")
```

```
## [1] "Definitions and Sources" "RUCA2020 Tract Data"
## [3] "Codebook"
```

```r
# Step 2: Read the correct sheet (update "SheetName" once you know it)
ruca_data <- read_excel("/Users/madelineratoza/Desktop/RUCA-codes-2020-tract.xlsx", sheet = "RUCA2020 T

# Step 3: Check column names to confirm the FIPS variable
colnames(ruca_data)
```

```
##  [1] "TractFIPS23"               "CountyFIPS23"
##  [3] "CountyCode23"              "CountyName23"
##  [5] "TractFIPS20"               "tract"
##  [7] "TractName20"               "CountyFIPS20"
##  [9] "CountyCode20"              "CountyName20"
## [11] "StateFIPS20"               "StateName20"
## [13] "UrbanAreaCode20"           "UrbanAreaName20"
## [15] "UrbanCore"                 "UrbanCoreType"
## [17] "PrimaryRUCA"               "PrimaryRUCADescription"
## [19] "PrimaryDestinationCode"    "PrimaryDestinationName"
## [21] "SecondaryRUCA"             "SecondaryRUCADescription"
## [23] "SecondaryDestinationCode" "SecondaryDestinationName"
## [25] "Population"                "LandArea"
## [27] "PopDensity"
```

```r
library(readxl)

# Load your RUCA file (adjust sheet number if needed)
ruca_data <- read_excel("/Users/madelineratoza/Desktop/RUCA-codes-2020-tract.xlsx", sheet = 2)
```

```r
# Check the column names
colnames(ruca_data)
```

```
##  [1] "TractFIPS23"               "CountyFIPS23"
##  [3] "CountyCode23"              "CountyName23"
##  [5] "TractFIPS20"               "tract"
##  [7] "TractName20"               "CountyFIPS20"
##  [9] "CountyCode20"              "CountyName20"
## [11] "StateFIPS20"               "StateName20"
## [13] "UrbanAreaCode20"           "UrbanAreaName20"
## [15] "UrbanCore"                 "UrbanCoreType"
## [17] "PrimaryRUCA"               "PrimaryRUCADescription"
## [19] "PrimaryDestinationCode"    "PrimaryDestinationName"
## [21] "SecondaryRUCA"             "SecondaryRUCADescription"
## [23] "SecondaryDestinationCode" "SecondaryDestinationName"
## [25] "Population"                "LandArea"
## [27] "PopDensity"
```

```r
library(readxl)
library(dplyr)
library(stringr)

# Load your data
ratio_data <- read_excel("/Users/madelineratoza/Desktop/Ratio.xls")
ruca_data  <- read_excel("/Users/madelineratoza/Desktop/RUCA-codes-2020-tract.xlsx", sheet = 2)

# Convert 'tract' to 11-character string
ratio_data <- ratio_data %>%
  mutate(tract = str_pad(as.character(tract), 11, pad = "0"))

ruca_data <- ruca_data %>%
  mutate(tract = str_pad(as.character(tract), 11, pad = "0"))

#  Keep only one record per tract (selecting the PrimaryRUCA)
ruca_unique <- ruca_data %>%
  select(tract, PrimaryRUCA) %>%
  distinct(tract, .keep_all = TRUE)

# Join
merged_data <- ratio_data %>%
  left_join(ruca_unique, by = "tract") %>%
  mutate(
    UrbanRural = case_when(
      PrimaryRUCA < 4 ~ "Urban",
      TRUE ~ "Rural"
    )
  )

# Check counts (should be about 6,896)
nrow(merged_data)
```

```
## [1] 6896
```

```r
# Summarize again
summary_table <- merged_data %>%
  group_by(UrbanRural) %>%
  summarize(
    n = n(),
    mean_ratio = mean(PopulationtoProviderZerotoOne, na.rm = TRUE),
    median_ratio = median(PopulationtoProviderZerotoOne, na.rm = TRUE),
    IQR_ratio = IQR(PopulationtoProviderZerotoOne, na.rm = TRUE),
    min_ratio = min(PopulationtoProviderZerotoOne, na.rm = TRUE),
    max_ratio = max(PopulationtoProviderZerotoOne, na.rm = TRUE)
  )

print(summary_table)
```

```
## # A tibble: 2 x 7
##   UrbanRural     n mean_ratio median_ratio IQR_ratio min_ratio max_ratio
##   <chr>      <int>      <dbl>        <dbl>     <dbl>     <dbl>     <dbl>
## 1 Rural       1162      1563.         1093     1690.      8        10510
## 2 Urban       5734      1763.         1141     2054.      4.48     11147
```

```r
library(ggplot2)

ggplot(merged_data, aes(x = UrbanRural, y = PopulationtoProviderZerotoOne)) +
  geom_boxplot() +
  scale_y_log10() +
  labs(
    title = "Distribution of Population-to-Provider Ratios by Rurality",
    x = "Rurality (RUCA Classification)",
    y = "Population-to-Provider Ratio (log scale)"
  ) +
  theme_minimal()
```

```
## Warning: Removed 44 rows containing non-finite outside the scale range
## (`stat_boxplot()`).
```

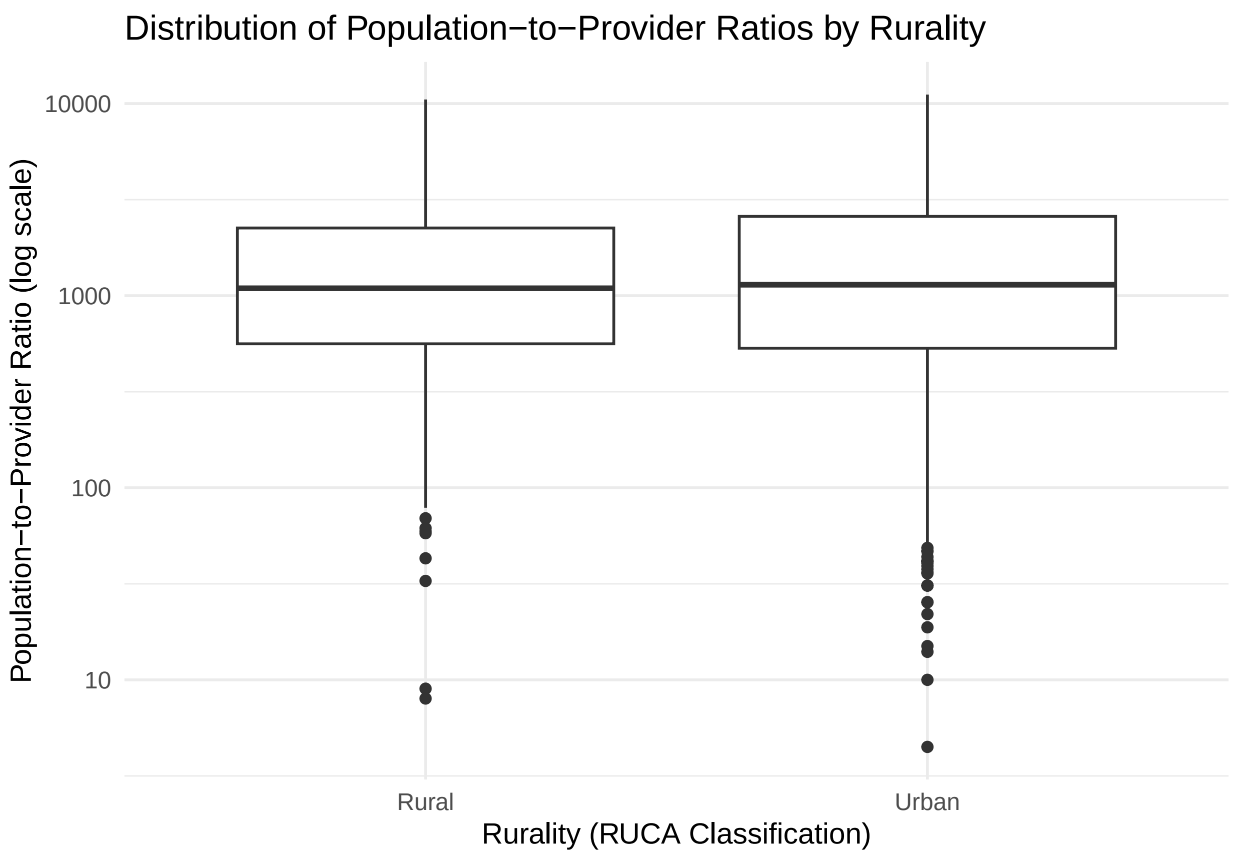


```r
library(ggplot2)

ggplot(merged_data, aes(x = UrbanRural, y = PopulationtoProviderZerotoOne)) +
  geom_boxplot() +
  labs(
    title = "Distribution of Population-to-Provider Ratios by Rurality",
    x = "Rurality (RUCA Classification)",
    y = "Population-to-Provider Ratio"
  ) +
  theme_minimal()
```

```
## Warning: Removed 44 rows containing non-finite outside the scale range
## (`stat_boxplot()`).
```

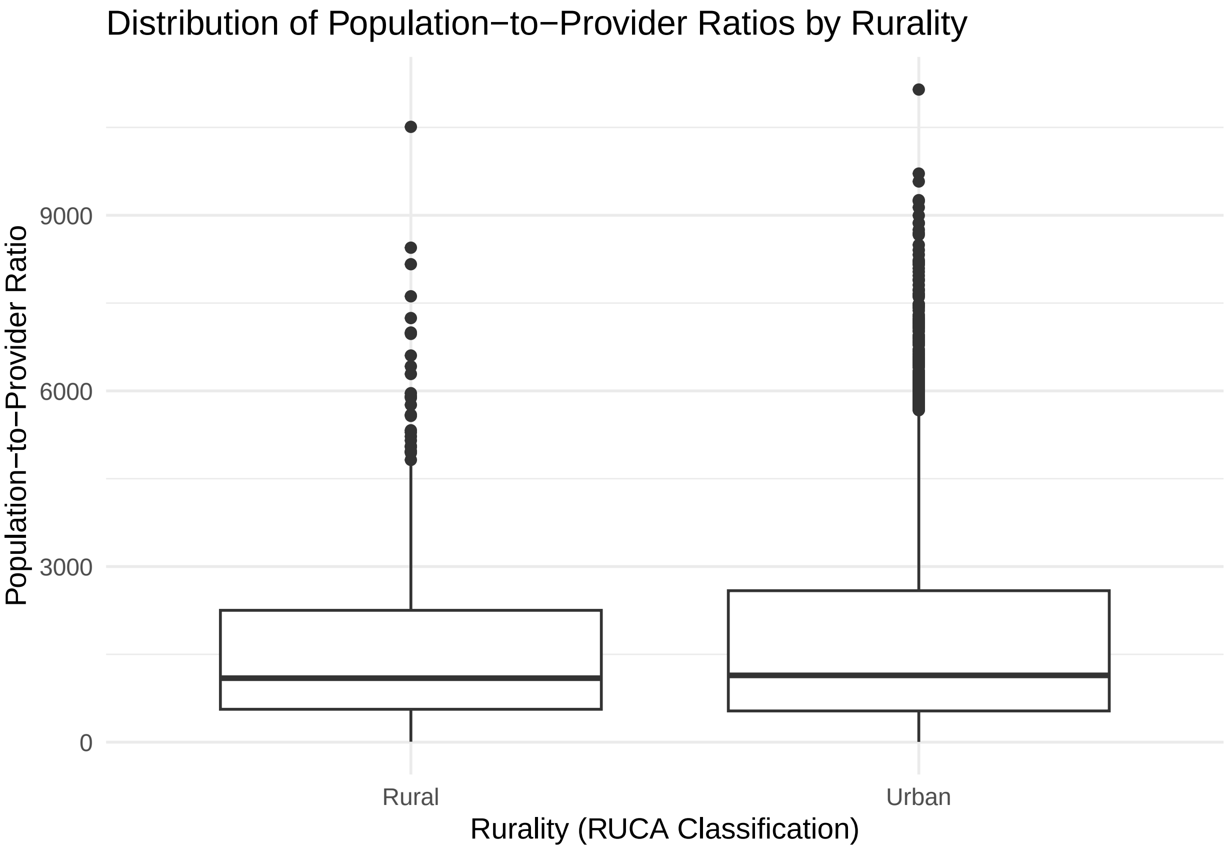

Distribution of Population−to−Provider Ratios by Rurality
Population−to−Provider Ratio
9000
6000
3000
0
Rural
Urban
Rurality (RUCA Classification)
