# Supplementary material for: Mapping the Availability of Rehabilitation Providers Using Public Licensure and Population Data for a Geographic Information System–Based Approach to Workforce Planning: Cross-Sectional Feasibility Study
Source: JMIR Form Res. 2025 Dec 23;9:e85025. doi: 10.2196/85025 (PMC12775756; doi:10.2196/85025)
Supplement: Multimedia Appendix 6 [file formative_v9i1e85025_app6.pdf]

Spatial Autocorrelation Report

Moran's Index 0.305672

z-score 40.287256

p-value 0.000000

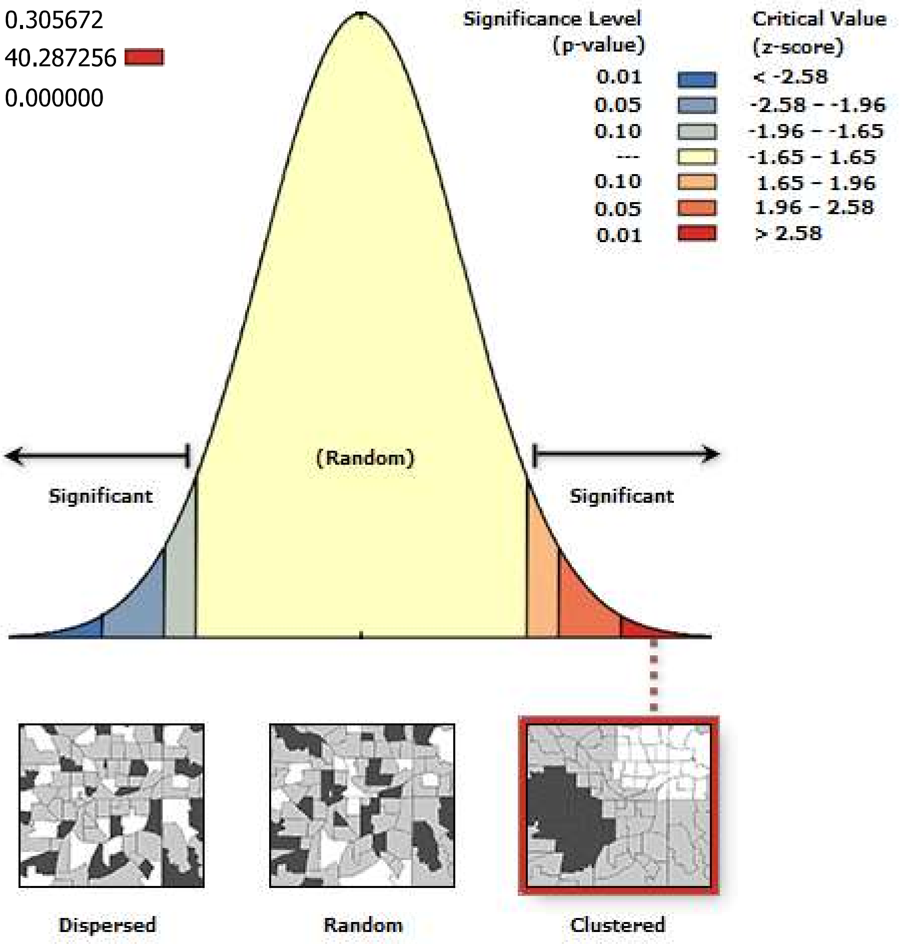

Given the z-score of 40.287256, there is a less than 1% likelihood that this clustered pattern could be the result of random chance.

Global Moran's I Summary

**Moran's Index**	0.305672
**Expected Index**	-0.000146
**Variance**	0.000058
**z-score**	40.287256
**p-value**	0.000000

Dataset Information

**Input Feature Class:**	TexasCensusTract_SpatialJoin11
**Input Field:**	POPULATIONTOPROVIDERZEROTOONE
**Conceptualization:**	CONTIGUITY_EDGES_ONLY
**Distance Method:**	EUCLIDEAN

**Row Standardization:**	True
**Distance Threshold:**	None
**Weights Matrix File:**	None
**Selection Set:**	False
